# Impact of the introduction of new vaccines and vaccine wastage rate on the cost-effectiveness of routine EPI: lessons from a descriptive study in a Cameroonian health district

**DOI:** 10.1186/1478-7547-9-9

**Published:** 2011-05-28

**Authors:** Cliford E Ebong, Pierre Levy

**Affiliations:** 1 Ngong District Health Service, Cameroon; 2 Université Paris Dauphine, LEDa-LEGOS, France

**Keywords:** EPI (Expanded Program of Immunization), cost-effectiveness, FIC (Fully Immunized Child), Excess vaccine wastage, Pentavalent vaccine (DPT-HB-Hib), new vaccines, Cameroon, Ngong

## Abstract

The Expanded Program of Immunization (EPI) offers services to the population free of charge but these activities are costly with the greatest part being the cost of vaccines. In spite of the growing international solidarity towards funding for immunization, the growing objectives continue to outweigh the available resources. It is therefore crucial for any immunization system to seek greater efficiency so as to optimize the use of available means in a bid to ensure sustainability. It is in this light that we carried out this study which aims to assess the productive efficiency of routine EPI for children aged 0 - 11 months with respect to the fixed and outreach vaccine delivery strategies in Ngong health district. The study is descriptive and cross-sectional. Data were collected retrospectively for all 16 health centers of the district that offered EPI services during the period February - May 2009.

The results show that:

• Only 62% of planned outreach immunization sessions were effectively carried out mainly due to limited funds for transportation and staff availability. Consequently vaccine coverage was low (BCG: 70.1%, DPT-HB-Hib 3: 55.5%) and less resources (43%) were used for this strategy which served 52% of the target population - a major blow to equity.

• The average cost per Fully Immunized Child (FIC) was 9,571 FCFA (19.22 USD) for the fixed strategy; 12,751 FCFA (25.61 USD) for the outreach and 10,718 FCFA (21.53 USD) with both strategies combined. These figures are high than those observed in many other African health districts. However, DPT-HB-Hib and yellow fever vaccines contributed to the increase as vaccines occupied 57% of the total cost. With DPT in lieu of DPT-HB-Hib the cost/FIC would be 6,046 FCFA (12.14 USD). Dropout rates too were high (28.1% for the fixed, 29.7% for outreach).

• The cost of vaccines wasted in excess of the national norm at the level of health centers was 595,532 FCFA (1,196.15 USD), an amount that could cover the vaccine cost for 122 FIC (7.6% of the FIC during the period). This was accounted for as follows: BCG 1.1%, OPV 1.4%, DPT-HB-Hib 72.7%, measles 5.3%, yellow fever 19.5%

• Therefore we suggest improved communication for EPI, the introduction of DPT-HB-Hib with liquid Hib and the effective implementation of planned outreach sessions.

## Background

Vaccines are costly and from many studies constitute a major burden for every immunization program [1,2]. With the introduction of the GIVS (Global Immunization, Vision and Strategy), immunization coverage objectives have been raised to reach more children (equity) and to cover more diseases. This involves the introduction of new vaccines and combinations which are generally more expensive. In this light, the Pentavalent vaccine, DPT-HB-Hib was introduced (in place of DPT) into the Expanded Program of Immunization (EPI) in Cameroon in February 2009, alongside the other traditional vaccines BCG, OPV, Measles and yellow fever (YF) vaccines.

**Figure 1:**
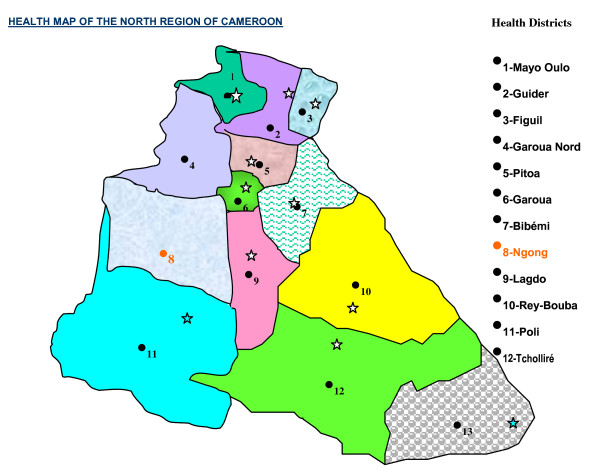
**The Map of the North region of Cameroon showing the health districts**.

**Figure 2:**
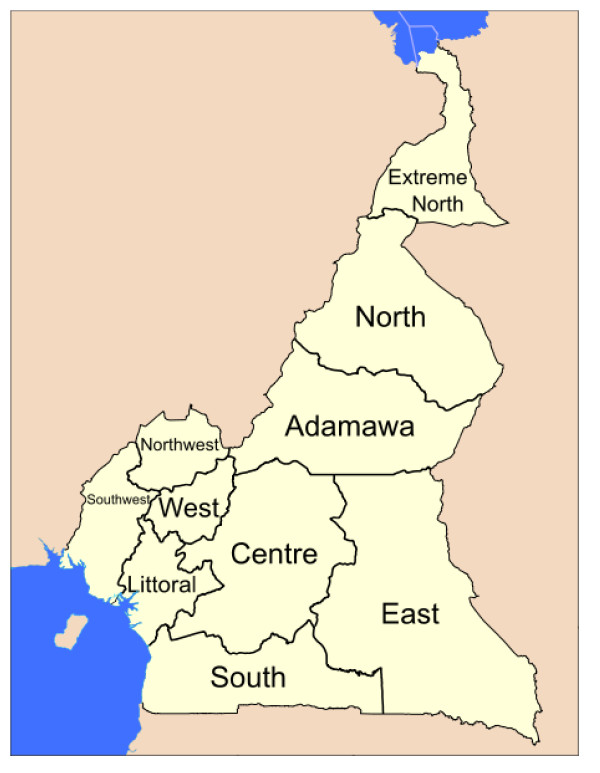
**The Map of Cameroon showing the ten regions**.

The Ngong health district in the North region of Cameroon (Figures 1 and 2) covers an area of 4,000 km^2 ^and served a total population of 143,238 in 2009, about half of them cattle raisers. There are 5,730 target children (aged 0-11 months) disseminated in 197 villages with an annual average target of 36 ± 57 children per village. The target for BCG (live births) was 6,446. Two vaccine delivery strategies are used for routine EPI: the fixed - at health facilities - and the outreach - for those who live more than 5 km from a health center (52%). The mobile strategy (requires teams to go out to villages situated farther than 20 km away with a vehicle and spend days to serve many villages) is not used for logistical reasons (most health centers do not even have a motorcycle) and staff numbers are limited. So teams prefer to go out to these villages by various means and return to the health center at the end of the day.

Unfortunately, resources for immunization do not follow proportionately the growing coverage objectives of EPI and the GIVS (Global Immunization, Vision and Strategy) in spite of the global solidarity towards funding (GAVI, WHO, UNICEF, among others). This raises concerns about the long term sustainability of the activities of the program. Therefore, it is of the utmost importance to improve efficiency so as to rationally use the available resources for the best possible results.

A health districts like Ngong with a large area and a sparse and very mobile population disseminated in many small villages, introduces both a high risk of vaccine wastage and a high risk of dropout. Furthermore, the top-down distribution of vaccines - from the central level to the regions, then to each health district, and then to health centers in proportion to approximate target populations - may worsen the direct impact of wastage on coverage and efficiency. This is because the quantity of vaccines is determined by the central level on the basis of administrative target populations, coverage objectives, wastage norms and a security margin. In a context of high population mobility, the target population is hard to master and vaccine wastage in excess of the national norm drastically increases the risk of vaccine stock-outs.

This study aimed to assess the impact of the introduction of 2-dose lyophilized DPT-HB-Hib and of vaccine wastage rate on the cost-effectiveness of EPI as well as to identify main explaining factors of such results. The primary objective of the study was to assess the productive efficiency of EPI for children aged 0-11 months with respect to vaccine delivery strategy - fixed and outreach - taking into account the differences between the two strategies in terms of vaccine coverage and vaccine wastage. The secondary objectives were to estimate the cost of excess vaccine wastage and to describe the various mechanisms of vaccine wastage but also to quantify the benefits of the open vial policy (reuse of open liquid vaccines within four weeks in the absence of contamination, expiration, Vaccine Vial Monitor color change and exposure to light).

## Materials and methods

This study was descriptive and cross-sectional. Data collection was done retrospectively in July and August for the period February to May 2009. All 16 health centers that offered EPI services were studied. Data on vaccine use were obtained by exploitation of vaccine usage forms, vaccine movement registers and monthly EPI reports. Costs for vaccines and consumables were estimated using the UNICEF-WHO price projections [3]. The unit prices used were 45 FCFA for BCG, 1,397 FCFA for Penta, 61 FCFA for OPV, 88 FCFA for measles vaccine, 326 FCFA for YFV, 38 FCFA for auto disable syringes, 60 FCFA for dilution syringes, and 299 FCFA for a safety box. The other costs (personnel, transport, EPI running, Social mobilization, short term training, maintenance, running the cold chain, amortization for buildings, 'rolling stock', and refrigerators) were obtained by interview, direct surface measurements of buildings, estimation of time spent for EPI by the staff involved and the exploitation of various documents (immunization sessions plan, tally sheets, staff pay vouchers, cash movement registers, receipts and building reception documents) at the level of health centers and the district health service.

The following shared health system costs were considered: personnel (generally polyvalent), buildings and the rolling stock (motorcycles and the district truck). The EPI cold chain was considered to be used 100% for the program. Personnel costs were estimated by multiplying their salary during the period by the proportion of their time spent for EPI activities and then adding to it any collation paid them. The activities considered include vaccination sessions, transportation of vaccines, reporting (immunization and disease surveillance), coordination meetings, supervision, and cold chain temperature monitoring and maintenance. It was estimated that vaccination sessions and supervision take 4 hours each and coordination meetings 8 hours.

Amortization costs for buildings, rolling stock and cold chain (refrigerators and the district deep freezer) during the study period of 4 months were estimated by dividing their respective costs by the amortization period - assumed 25 years for buildings and 5 years for 'rolling stock' and cold chain. This was later divided by 3 (4 months/12) and then multiplied by the proportion of use for EPI. Both time and space were considered for buildings (number of days a week and proportion of surface used). Time, space, and other resources spent for activities not specific to either the fixed or outreach strategy, were shared to the two according to target population.

The data, collected on a data collection spreadsheet made for the purpose, were analyzed with Excel software. The main indicator of effectiveness was the number of fully immunized children (FIC, children who received all 3 doses of DPT-HB-Hib) and the main indicator of efficiency was the cost per FIC. The costs of the district health service were shared to the health centers by strategy in proportion to target population covered. Other indicators used are the average cost per dose used (total costs/total doses used) and the average cost per dose administered (total costs/total doses administered - what it takes to administer a dose). Costs are expressed in local currency (FCFA) as well as in USD using an exchange rate of 497.87 FCFA for 1 USD (official WHO/UN April 2009).

## Results

### 1. Vaccine coverage

**Figure 3:**
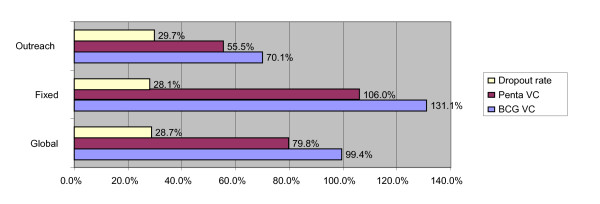
**Vaccination coverage for the district and dropout rate for BCG - Penta 3**.

**Figure 4:**
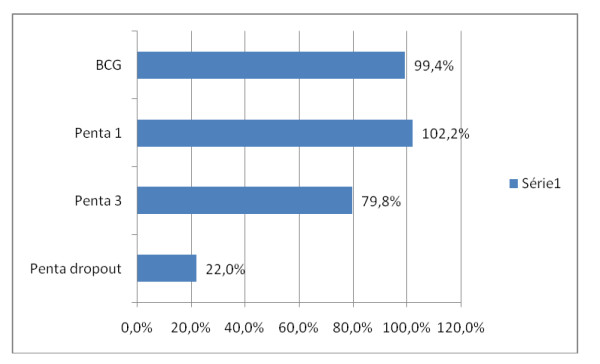
**The Global (fixed + outreach) vaccine coverage for BCG, Penta 1 and Penta 3 and specific dropout rate for Penta**.

Only 62% of planned outreach vaccination sessions were effectively carried out against 95% for the fixed. Of 2,271 live births expected during the period, 2,258 doses of BCG were administered thus reaching a coverage of 99.4%. Of 2,019 children aged 0 - 11 months expected 1,610 doses of Penta 3 (FIC) were administered - 1,029 by the fixed strategy against 581 by outreach. This gives a 79.8% coverage (Figures 3 and 4). Penta 1 coverage was 102.2% and the specific dropout rate (Penta 1 to Penta 3) was 22.0%. Vaccine coverage above 100% is common. It is calculated on the basis of administrative data which are not usually up-to-date in this context of very rampant human movement. It was generally higher for the fixed strategy as compared to the outreach but the BCG - Penta 3 dropout rates were very similar (figure 3).

### 2. EPI Costs

Recurrent costs made up for 92.2% of total EPI costs with up to 61.1% for vaccines and consumables (table 1), 57% for vaccines alone. This is even greater for the fixed strategy. Penta vaccine accounted for 78% of the total cost of vaccines and consumables (8,242,300 FCFA). This is due to its high cost and the need for up to 3 doses per FIC. Whereas personnel is known for being the largest cost category, generally accounting for more than half of the total cost [1,2], it accounts for only 21% of total cost in the present study. In fact the government health staff situation was so poor in Ngong health district that health facilities resort to the use of unpaid or poorly paid staff. This is further accentuated by a high cost for vaccines.

**Table 1:** EPI costs and their structure for the district

COST POST	COST BY POST (F CFA)			COST STRUCTURE		
	
	Fixed	Outreach	Global	Fixed	Outreach	Global
VACCINES/CONSUMABLES	6,374,600	4,163,584	10,538,184	64.7%	56.2%	61.1%
TRANSPORT/FUEL	319,895	543,705	863,600	3.2%	7.3%	5.0%
MAINTENANCE	31,713	121,687	153,400	0.3%	1.6%	0.9%
SALARIES/COLLATIONS	2,108,912	1,529,536	3,638,448	21.4%	20.6%	21.1%
SOCIAL MOBILIZATION	17,800	34,330	52,130	0.2%	0.5%	0.3%
SHORT TERM TRAINING	11,542	12,458	24,000	0.1%	0.2%	0.1%
PROGRAMME RUNNING	55,256	41,874	97,130	0.6%	0.6%	0.6%
COLD CHAIN RUNNING	262,061	288,899	550,960	2.7%	3.9%	3.2%

AMORTIZATION	666,479	672,302	1,338,781	6.8%	9.1%	7.8%

**TOTAL COST**	**9,848,259**	**7,408,374**	**17,256,633**	**100.0%**	**100.0%**	**100.0%**

### 3. Efficiency of EPI in the District

**Figure 5:**
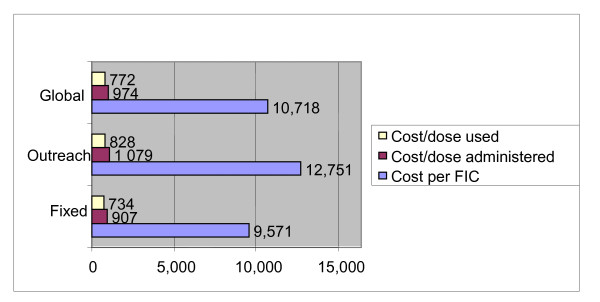
**The Average cost per FIC, Dose Administered and Dose Used in FCFA**.

The average cost/FIC was 9,571 FCFA (19.22 USD) for the fixed strategy and 12,751 FCFA (25.61 USD) for the outreach (figure 5). The global average cost/FIC for the district was 10,718 FCFA (21.53 USD). Disaggregated results show a 5-time variation among health centers, ranging from 7,208 FCFA (14.48 USD) at Ngong to 36,101 FCFA (72.51 USD) at Bangli for the fixed strategy (Table 2). The Boumedje health center that started activity during the period ad that only implemented one round of outreach in four months had a total cost of 163,641 FCFA (328.68 USD) for the outreach strategy and no FIC since no dose of Penta 3 was administered. With the cost of vaccines and consumables ignored, the cost per FIC for the district remains higher with the outreach strategy, and with the cost for DPT introduced in lieu of Penta we notice a 77% increase due to Penta (Table 3).

**Table 2:** Cost per fully immunized child by health center and by vaccine delivery strategy after distributing district service costs in proportion to target population

Health center	Total cost (FCFA)		*Cost per FIC (FCFA)*	
	
	Fixed	Outreach	Fixed	Outreach
Bangli	252,706	280,968	36,101	14,048
Nakong	766,584	523,730	11,273	20,949
Djalingo	935,537	1,400,112	7,996	8,001
Babla	471,313	288,826	8,569	10,697
Kismatari	389,303	202,815	29,946	14,487
Djefatou	482,438	0	11,487	/
Karewa	236,879	140,397	12,467	10,800
L Tchitta	362,935	439,661	20,163	14,655
Ndjola	898,716	0	10,573	/
L Massa	528,625	427,479	9,974	8,724
Ngong	1,787,465	1,012,914	7,208	14,470
Boumedje	495,491	163,641	7,507	163,641/0
Tcheboa	688,132	430,507	8,822	18,718
S Ngal	564,169	0	7,836	/
Touroua p.	591,052	1,315,442	8,209	16,240
St Vincent	396,912	781,884	24,807	14,479
**Total**	**9,848,259**	**7,408,374**	**9,571**	**12,751**

**Table 3:** Cost per FIC for the district under special considerations

Condition	Cost per FIC		
	
	Fixed strategy	Outreach strategy	Global
With Penta	9,571 FCFA (19.22 USD)	12,751 FCFA (25.61 USD)	10,718 FCFA (21.53 USD)

With DPT in lieu of Penta	5,098 FCFA (10.24 USD)	7,726 FCFA (15.52 USD)	6,046 FCFA (12.14 USD)

Vaccines and consumables ignored	3,376 FCFA (6.78 USD)	5,585 FCFA (11.22 USD)	4,173 FCFA (8.38 USD)

### 4. Vaccine wastage

The wastage rate was generally lower in the fixed strategy except for OPV (table 4). Whereas for traditional antigens, such as BCG and OPV, the district wastage rate was within the accepted range, this was not the case for more expensive and newer vaccines, mainly Penta and YFV. However it was much lower than previously reported in similar studies in other African health districts [4-6]. An inquiry into the causes of wastage showed that 98% of vaccine doses were lost after the vial was opened as already noted in a previous study in Benin [4]. No vaccines were lost at the district level. One reason why OPV wastage is within accepted range is that the open vial policy is applied in 9 of the 16 health centers and 72.8% of all open vials of OPV and tetanus toxoid (for pregnant women) produced were reused at least once, in line with previously published results [4].

**Table 4:** Wastage rate by strategy and antigen for the district of Ngong

Strategy	Antigens				
	
	BCG	PENTA	OPV	Measles	YFV
Fixed	37.7%	6.8%	15.7%	27.2%	27.2%
Outreach	50.2%	7.1%	9.6%	38.8%	38.8%

**Global**	**43.0%**	**6.9%**	**13.5%**	**32.6%**	**32.6%**

**National norm**	**< 50%**	**< 5%**	**< 25%**	**< 25%**	**< 25%**

The values of vaccine doses wasted in excess of the national norm (excess vaccine wastage) at individual health centers sum up to 301,264 FCFA (605.11 USD) for the fixed strategy and 294,268 FCFA (591.05 USD) for the outreach (Table 5). These two amounts equate to the total cost for vaccines for 122 FIC (7.6% of FIC during the period). The cost structure of this excess vaccine wastage was as follows: BCG 1.1%, VPO 1.4%, Penta 72.7%, Measles 5.3%, YFV 19.5%. This clearly shows that newer and much more expensive vaccines such as Penta have a dramatic impact on the cost of wastage and put to the forefront the need for a better control and increased effectiveness of delivery services.

**Table 5:** Sum total of excess wastage at individual health center level and by delivery strategy

Antigen	Accepted wastage rate	Excess wastage (doses)		Cost of excess wastage			
		
		Fixed	Outreach	Fixed	Outreach	Global	Percentage
BCG	50%	148	0	6,660	0	6,660	1.1

OPV	25%	121	17	7,381	1,037	8,418	1.4

Penta	5%	165	145	230,505	202,565	433,070	72.7

Measles	25%	137	219	12,056	19,272	31,328	5.3

YFV	25%	137	219	44,662	71,394	116,056	19.5

**Total**		**708**	**600**	**301,264**	**294,268**	**595,532**	**100**

## Discussion

One of the main findings of this study is that vaccine coverage is lower for the outreach strategy than for the fixed while dropout rate is high for both strategies. Reasons for such a result lie in inadequate sensitization, high population mobility and low implementation of planned outreach vaccination sessions, the latter being due to limitations in funds generation or allocation at the health center level for the transportation of teams. The desire to moderate vaccine wastage in a setting with numerous small villages where attendance at immunization sessions is usually low creates missed chances as staffs often postpone the administration of an antigen until more children are present. These are missed chances. Nevertheless vaccine wastage was higher than the national norm and the cost of excess wastage amounted to the vaccine cost for 122 additional FIC (7.6% of FIC during the study period). The most important part of this excess cost was due to the loss of newer, expensive vaccines (Penta and YFV). Whereas wastage occurs essentially after the opening of vials (98%), the correct application of the open vial policy in a majority of health centers explains why OPV wastage was contained within accepted limits for the district.

Concerning EPI costs, it appears that recurrent costs are very predominant and are largely due to the cost of vaccines (Penta, YFV). Personnel costs accounted for a smaller part due to the use of unpaid staff and the weight of vaccine costs. Moreover, training and sensitization/social mobilization represent negligible cost categories. This can explain the low coverage and high dropout of the target population especially for the outreach strategy where fewer resources (43%) are used for a greater portion of the population (52%).

As a consequence, the efficiency of EPI is suboptimal. The average cost per FIC (USD 21,53) is greater than figures obtained in other African health districts [7-9]. This could be partly due to methodological differences concerning the estimation of personnel costs. The latter are often restricted to incremental costs incurred by outreach strategies (*perdiems or collation*) and not the full costs imputable to vaccination as in the present study. Two main factors contributing to true suboptimal efficiency are high dropout rates caused by inadequate sensitization/social mobilization and a difficult demography. But this result should also be viewed in the light of the introduction of Penta, a more expensive vaccine. These differences could explain why numerous studies across the world found an average cost per FIC which was very variable depending on the country of interest and the reference year. Studies conducted during the 1980s found a range from USD 5 to USD 15 per FIC while later studies found a range from USD 10 to USD 20 and even higher [10,11].

The 5-time variation in cost per FIC amongst health centers can be analyzed as follows. The cost per FIC is lowest in Ngong and Djalingo that serve dense semi-urban populations and that have high numbers of FIC, a major determinant of cost per FIC [12]. In contrast, in Bangli where for about the same fixed costs coverage is low and dropout high because of poor general use of the health center, the cost per FIC is 5-times higher for the fixed strategy. Just one nurse serves in this health center and the nearby population, in addition to being rural (with low literacy rate) and requiring even more sensitization, has a reputation of being very exigent.

Vaccine wastage in excess of the national norm is highest for penta. This may signify that due to its high cost, an overambitious threshold level of wastage (5%) was set. When the health centers are considered individually, even antigens with a normal wastage rate at the district level have excess wastage.

## Conclusions

- Efficiency of EPI is lower with the outreach strategy in Ngong health district because of poor implementation of planned immunization sessions, a difficult population settlement situation and inadequate social mobilization. However, the strategy needs to be reinforced as it improves equity and reduces indirect costs of immunization incurred by mothers who live in distant localities.

- There is a 5-time variation in cost per FIC amongst health centers, mainly due to low coverage and high dropout in poorly used health centers with less dense population. This projects the role of the number of FIC in determining the cost per FIC

- Excess vaccine wastage has a high cost when expensive vaccines are used, especially if the open vial policy cannot be applied as with lyophilized Hib vaccine, hence the need to resort to a presentation with liquid Hib. This would encourage vaccination staff to readily open a vial to immunize a child at every opportunity, given that the co-administered vaccine, OPV, is also liquid and the open vial policy applies.

## Abbreviations used

BCG: Bacille Calmette-Guerrin; DPT: Diphtheria, pertussis and tetanus vaccine; DPT-HB-Hib: Diphtheria, pertussis, tetanus, hepatitis B and Haemophilus influenza type b vaccine (Pentavalent vaccine or Penta); EPI: Expanded Program of Immunization; EPIVAC: Epidemiology, Vaccinology, and Management program; FCFA: African Franc, FIC: Fully Immunized Child; GIVS: Global Immunization, Vision and Strategy; OPV: Oral Poliomyelitis Vaccine, UN: The United Nations; UNICEF: United Nations International Children's Emergency Fond; USD: United States Dollar; WHO: The World Health Organization;, YFV: Yellow fever vaccine

## Competing interests

The authors declare that they have no competing interests.

## Authors' contributions

CEE Conceived the study and performed the initial study design, the data collection and analysis and the initial write-up

PL revised the study design, ensured the supervision, did the final corrections of the manuscript

Both authors read and approved the final manuscript

## Authors' information

C.E.E.: MD, DIU 3^e ^cycle Management/applied vaccinology (EPIVAC)

*District Medical Officer, Ngong, Cameroon, e-mail: *http://cliffebong@yahoo.com

P. L.: PhD in Economics

*Assistant Professor, Université Paris Dauphine, LEDa-LEGOS, France, e-mail: *http://pierre.levy@dauphine.fr
